# Parallel and Sequential Pathways of Molecular Recognition of a Tandem-Repeat Protein and Its Intrinsically Disordered Binding Partner

**DOI:** 10.3390/biom11060827

**Published:** 2021-06-01

**Authors:** Ben M. Smith, Pamela J. E. Rowling, Christopher M. Dobson, Laura S. Itzhaki

**Affiliations:** 1 Department of Pharmacology, University of Cambridge, Tennis Court Road, Cambridge CB2 1PD, UK; bms40@cantab.net; 2 Department of Chemistry, University of Cambridge, Lensfield Road, Cambridge CB2 1EW, UK; cmd44@cam.ac.uk

**Keywords:** repeat protein, tandem-repeat protein, intrinsic disorder, intrinsically disordered protein, fuzzy complex, protein-protein interactions, protein folding, armadillo repeat, beta-catenin, TCF

## Abstract

The Wnt signalling pathway plays an important role in cell proliferation, differentiation, and fate decisions in embryonic development and the maintenance of adult tissues. The twelve armadillo (ARM) repeat-containing protein β-catenin acts as the signal transducer in this pathway. Here, we investigated the interaction between β-catenin and the intrinsically disordered transcription factor TCF7L2, comprising a very long nanomolar-affinity interface of approximately 4800 Å^2^ that spans ten of the twelve ARM repeats of β-catenin. First, a fluorescence reporter system for the interaction was engineered and used to determine the kinetic rate constants for the association and dissociation. The association kinetics of TCF7L2 and β-catenin were monophasic and rapid (7.3 ± 0.1 × 10^7^ M^−1^·s^−1^), whereas dissociation was biphasic and slow (5.7 ± 0.4 × 10^−4^ s^−1^, 15.2 ± 2.8 × 10^−4^ s^−1^). This reporter system was then combined with site-directed mutagenesis to investigate the striking variability in the conformation adopted by TCF7L2 in the three different crystal structures of the TCF7L2–β-catenin complex. We found that the mutation had very little effect on the association kinetics, indicating that most interactions form after the rate-limiting barrier for association. Mutations of the N- and C-terminal subdomains of TCF7L2 that adopt relatively fixed conformations in the crystal structures had large effects on the dissociation kinetics, whereas the mutation of the labile sub-domain connecting them had negligible effect. These results point to a two-site avidity mechanism of binding with the linker region forming a “fuzzy” complex involving transient contacts that are not site-specific. Strikingly, the two mutations in the N-terminal subdomain that had the largest effects on the dissociation kinetics showed two additional phases, indicating partial flux through an alternative dissociation pathway that is inaccessible to the wild type. The results presented here provide insights into the kinetics of the molecular recognition of a long intrinsically disordered region with an elongated repeat-protein surface, a process found to involve parallel routes with sequential steps in each.

## 1. Introduction

β-catenin fulfils two important functions in vivo, the first of which is as the signal transducer in the canonical Wnt signalling pathway [1]. Upon Wnt stimulation, β-catenin translocates to the nucleus, where it binds the T-cell factor/lymphoid enhancer-binding factor (TCF/LEF) family of transcription factors [2] and associates with a number of other proteins to form the Wnt enhanceosome [3], thus leading to transcription initiation and elongation, as well as histone and chromatin modification [4,5], resulting in the transcription of genes that are of developmental importance and those involved in tissue homeostasis. In the absence of a Wnt signal, cytosolic β-catenin is continuously synthesised and then sequestered and targeted for proteasomal degradation by a multi-protein complex called the β-catenin destruction complex (BDC) that forms biological condensates [6]. The BDC comprises five different proteins: two structural proteins (axin and adenomatous polyposis coli (APC)), two kinases (glycogen synthase kinase 3β (GSK3β) and casein kinase 1α (CK1α)), and protein phosphatase 2A (PP2A). In the BDC, β-catenin is hyperphosphorylated at its N-terminal disordered region by the combined action of GSK3β and CK1α [7], thus allowing it to be recognised and ubiquitinated by the E3 ubiquitin ligase β-TrCP [8] and subsequently degraded by the proteasome. The second function of β-catenin is as an adaptor that mediates cell–cell adhesion at adherens junctions, where β-catenin binds to the intracellular domain of the cadherin family of proteins [1,9,10,11]. The deregulation of Wnt signalling has been implicated in numerous types of cancer [12], including colorectal and hepatocellular cancers. Additionally, the disruption of β-catenin binding at adherens junctions is also cancer-promoting [13]. In addition to being inherently pro-metastatic through the weakening of cell–cell adhesion [14], adherens junction-associated β-catenin constitutes a significant cellular pool of β-catenin [15], the release of which can result in its nuclear accumulation and unregulated gene activation [16].

**Figure 1:**
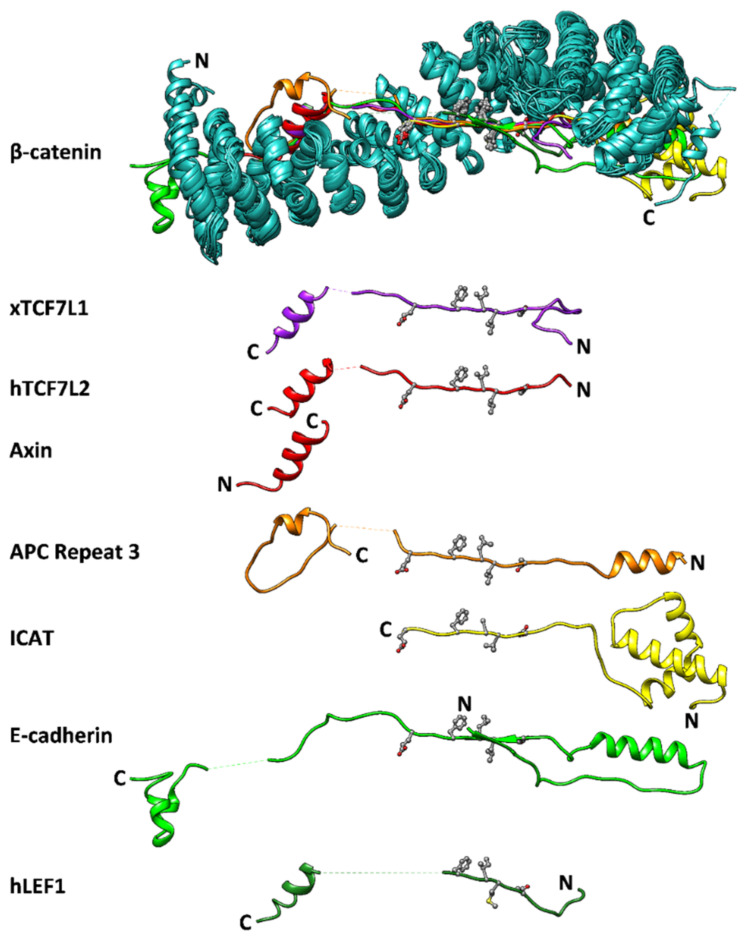
Interactions of β-catenin and its partner proteins. Schematic showing the superposition of the crystal structures of β-catenin (cyan) in complex with its seven binding partners (various colours), as visualised in UCSF Chimera [24]: *Xenopus* TCF7L1 (PDB ID 1G3J, [20] human Tcf4) (2GL7, [25] axin (1QZ7, [26])), APC third repeat (1TH1, [23]), ICAT (1LUJ, [27]), the cytoplasmic domain of E-cadherin (3IFQ, [28]), and human Lef-1 (3OUW, [29]). Disordered regions that are not visible in the crystal structures are denoted by a dashed line. All partner proteins, except for axin, bind in an antiparallel configuration relative to β-catenin. The consensus binding motif of β-catenin binding partners is DxΦΦxΩx_2–7_E—where Φ indicates a hydrophobic residue and Ω indicates an aromatic residue these residues are shown.

β-catenin has a ~530-residue central domain of 12 armadillo (ARM) tandem repeats flanked at each end by ~100-residue disordered regions [17,18]. The twelve imperfect ARM repeats linearly stack to form a right-handed superhelix of helices. The third helix of each ARM repeat lines the groove formed by the superhelix and is enriched in positively charged residues [17] Thus, the repeat array creates an elongated surface for β-catenin’s negatively-charged, intrinsically-disordered binding partners, APC, E-cadherin and the TCF/LEF family of transcription factors that wrap around it [19,20,21,22,23], (Figure 1).

The target genes for the canonical Wnt pathway have a conserved sequence that binds to members of the TCF/LEF family of transcription factors, of which transcription factor 7-like 2 (TCF7L2, also known as Tcf4) has been the most widely studied to date [30,31]. TCF7L2 mutations are implicated in many types of cancers [32] and have been shown to promote the migration and invasion of human colorectal cancer cells [33,34,35]. Single nucleotide polymorphisms within the TCF7L2 gene are also a genetic biomarker for an increased risk of both type 2 and gestational diabetes [36,37]. TCF7L2 is like many mammalian transcription factors in having an intrinsically disordered region (IDR). The absence of fixed tertiary structure is thought to give proteins conformational plasticity and enable them to bind to a number of different macromolecules in response to physiological needs [38,39,40,41,42,43,44]. Given their capacity to adjust to any given binding partner, the range of structures adopted by IDRs upon complex formation is unsurprising. Broadly, these can be divided into static complexes [45], where the IDR is ordered (and visible in X-ray crystal structures), and dynamic or “fuzzy” complexes, where the IDR retains a significant proportion (sometimes all) of its disorder in the complex [46,47,48]. IDP binding mechanisms are equally varied, and they have been grouped into four distinct classes: simple two-state binding, avidity, allovalency, and fuzzy binding [49]. Even in the more simple case of two-state binding, association can involve conformational selection or folding upon binding [50,51,52,53,54].

**Figure 2:**
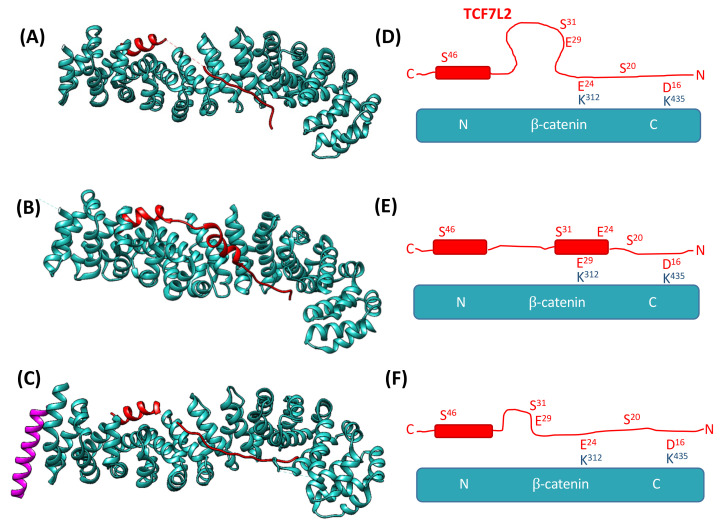
Schematics showing the crystal structures of the β-catenin–TCF7L2 complex. (**A**–**C**) show the structures of TCF7L2 (red) in complex with β-catenin (cyan) obtained by Poy et al. [22], Graham et al. [57], and Sampietro et al. [55], respectively. Structure (**C**) also has a peptide from the bound protein BCL9 (purple). (**D**–**F**) are schematics highlighting the different conformations of TCF7L2 between the three structures and indicating the positioning of the key contact residues within each structure. Images (**A**–**C**) were created using UCSF Chimera [24] from PDB 1JPW [22], 1JDH [55], and 2GL7 [25], respectively.

Intriguingly, there are three crystal structures of TCF7L2 in complex with the ARM repeat domain of β-catenin, with TCF7L2 adopting different conformations and making different β-catenin contacts in each (Figure 2) [20,22,25,55], though the Poy structure has poor refinement. In addition, there are significant portions of missing residues, and different regions are resolved in the different structures, suggesting that TCF7L2 is highly dynamic in complex with β-catenin. Similar behaviour has been observed in the crystal structures of other β-catenin complexes, providing tantalising hints to the underlying mechanisms of recognition [10,21,23,26,27,28,56]. Most recently, a single-molecule study by Hofmann and co-workers on the interaction between β-catenin and E-cadherin revealed a rugged energy landscape of E-cadherin with many shallow minima, a fuzzy complex, and a mechanism of intrachain diffusion of E-cadherin on β-catenin [57]. Here, we used site-directed mutagenesis and kinetic analysis to explore the significance of this plasticity in the β-catenin–TCF7L2 complex and to define the mechanism of association between the two proteins.

## 2. Materials and Methods

### 2.1. Molecular Biology and Protein Expression and Purification

Mutations in the β-catenin binding fragment of TCF7L2 (1–54 amino acids) were introduced by site directed mutagenesis. The plasmid encoding the ARM repeat domain of human β-catenin (residues 134–671) was a kind gift from Prof. W.I. Weis. All proteins were expressed in *E. coli* C41 cells [58]. Transformed cells were grown in 2TY with appropriate antibiotic until an OD600 of 0.6 at 37 °C was reached. Then the temperature was lowered to 25 °C, and protein expression were induced with 0.2 mM IPTG. After a further 18 h, the cells were harvested at 5000 g for 7 min at 4 °C. The cells were resuspended in 50 mM Tris-HCl buffer pH 7.5, 150 mM NaCl, and 1 mM DTT containing protease inhibitors and lysed using an Emusiflex C5 (Avestin, Ottawa, ON, Canada) at 10,000 psi. The lysed cells were centrifuged at 35,000× *g* for 35 min at 4 °C.

The GST-tagged β-catenin supernatant was incubated with glutathione-Sepharose 4B (Cytiva, Little Chalfont, Bucks, UK) and washed to remove unbound proteins. The GST tag was cleaved from the target protein on resin using thrombin, and the protein was eluted. β-catenin was further purified using a Mono-Q column (Cytiva, Little Chalfont, Bucks, UK) equilibrated in a 50 mM Tris-HCl buffer pH 8.9, 50 mM NaCl, and 1 mM DTT. β-catenin was eluted with a linear NaCl gradient to 1 M NaCl. Fractions containing greater than 95% β-catenin were pooled snap-frozen and lyophilised and stored at −80 °C.

The His-tagged TCF7L2 constructs were affinity-purified using Ni-NTA agarose (Qiagen, Manchester, UK), and the tagged constructs were cleaved from the protein using thrombin. The TCF7L2 was further purified on a HiLoad 26/600 Superdex 75 pg (Cytiva, Little Chalfont, Bucks, UK) equilibrated in PBS (phosphate buffer saline) containing 1 mM DTT. The fractions corresponding to A_215_ peaks were analysed by SDS-PAGE, and the fractions containing TCF7L2 at greater than >95% purity were flash-frozen with liquid nitrogen and stored at −80 °C until use.

The identities of all proteins were confirmed by MALDI mass spectrometry performed by Dr. Len Packman. The protein concentration of β-catenin was calculated from its extinction coefficient obtained using ProtParam [59] at 280 nm, and TCF7L2 protein concentration was measured using the Pierce™ BCA Protein Assay Kit (ThermoFisher Scientific, Waltham, MA, USA) or by ion-exchange-ninhydrin analysis performed by Dr. Peter Sharratt (PNAC Facility, Department of Biochemistry, University of Cambridge, Cambridge, UK).

### 2.2. Fluorescent Labelling of TCF7L2

The maleimide functional group reacts with sulfhydryl groups via nucleophilic conjugation addition and was used to label the proteins at a cysteine residue. A 500 µL aliquot of a TCF7L2 (1–54) construct containing a cysteine residue was buffer-exchanged into PBS. A stock solution of 20 mM fluorescein maleimide in 100% DMSO was added at a 5-times molar excess and incubated at 37 °C in the dark for 1 h. Excess fluorescein maleimide was removed by the method of Vivès and Lebleu, [60]. In brief, a 10× volume of acetone, pre-chilled to −20 °C, was added to the labelling reaction mixture, mixed by vortex, and incubated at −20 °C for 30 min. Fluorescein and its derivatives are very soluble in acetone, whereas peptides and proteins are insoluble. The addition of acetone to 90+% caused the protein to precipitate out of solution, while the unreacted fluorescein maleimide remained in solution. The reaction mixture was centrifuged at 15,000× *g* at 4 °C for 10 min to collect the precipitated and labelled protein, and then the supernatant was removed and the pellet was resuspended in PBS. This acetone wash process was repeated twice more, but the precipitated protein pellet was not resuspended on the final wash. The final precipitated protein pellet was left at room temperature and open to the air for 16 h to ensure that any residual acetone had evaporated, after which the desiccated and labelled TCF7L2 was stored at 4 °C for up to 5 days without a loss of activity.

### 2.3. Isothermal Titration Calorimetry (ITC)

ITC measurements were performed using the VP-ITC calorimeter (Malvern Pananalytical, Malvern, UK). Both β-catenin and TCF7L2 constructs were stored as lyophilised powders after labelling. To ensure matched buffers, lyophilised β-catenin was resuspended in ultra-pure water, the buffer was exchanged into PBS, 1 mM DTT, and the TCF7L2 was resuspended in the same buffer. Unlabelled TCF7L2 was washed with acetone three times, as previously described, to produce a lyophilised pellet. β-catenin was used at a concentration of 2–4 µM in the cell, and TCF7L2 was used at 6–100-fold higher concentration in the syringe (18–40 µM). Titrations of TCF7L2 into the buffer and the buffer into β-catenin were performed as controls. All experiments were performed at 30 °C. Data were fitted the Origin software package supplied with the instrument using the one-site binding equation. Data is shown in Supplementary Materials.

### 2.4. Kinetic Experiments

Stopped-flow spectroscopy was performed using an SX-19 Stopped flow fluorimeter (Applied Photophysics, Surrey, UK) in fluorescence mode. The excitation and emission wavelengths were set to 495 and 519 nm, respectively, and a 515 nm long-pass filter was used to improve the signal to noise ratio. All proteins were prepared in a fresh PBS buffer, 1 mM DTT, and the experiments were performed at 15 °C. The β-catenin concentrations were at least ten times higher than that of the TCF7L2 to ensure pseudo first-order conditions; a fixed concentration of a fluorescent-labelled TCF7L2 (10 nM) construct was rapidly mixed in a 1:1 volume ratio, with varying concentrations of unlabelled β-catenin (between 100 nM and 1000 nM), and the change in fluorescence intensity was recorded. For dissociation experiments, a complex of labelled TCF7L2 and β-catenin was pre-formed by mixing the two proteins in a 1:1 molar ratio (200 nM) and incubating them at 15 °C for 1 h in the dark to reach equilibrium. The pre-formed complex was then mixed in a 1:1 volume ratio with 50-times molar excess of unlabelled wild-type TCF7L2 (10 μM), and the change in fluorescence intensity was measured. A minimum of three traces was collected and averaged. The average was plotted using GraphPad Prism 6 (GraphPad Software, Ltd., San Diego, CA, USA) and fitted to either a single exponential phase or the sum of two exponential phases.

For the association experiments, the observed rate constants, *k_obs_*, were plotted against the concentration of β-catenin and *k_on_* calculated from the linear fit:

(1) $$k_{obs}=k_{on}\left[\beta \mathrm{catenin}\right]+c$$

For the slow dissociation kinetics, fluorescence spectroscopy was performed using a LS55 Luminescence Spectrometer (PerkinElmer, Waltham, MA, USA) using a cell of pathlength 10 mm. The excitation and emission wavelengths were set to 495 and 519 nm, respectively; both the excitation and emission shutter width were 5 nm; and the photomultiplier tube was set at 650 V. All proteins were prepared in a fresh 1× PBS buffer with 1 mM DTT, and the experiments were performed at 15 °C, except when otherwise stated. A complex of labelled TCF7L2 and β-catenin was pre-formed by mixing to two proteins in a 1:1 molar ratio and incubating them at 15 °C for 1 h in the dark to reach equilibrium. The pre-formed complex was then mixed in a 1:1 ratio with 50-times molar excess unlabelled WT TCF7L2, and the change in fluorescence intensity was measured. The traces were fitted as described above.

## 3. Results

### 3.1. The Association of TCF7L2 and β-Catenin Is Monophasic, and the Dissociation of the Complex Is Biphasic

In order to study the kinetics of TCF7L2–β-catenin complex formation and dissociation, we used a recombinant fragment of TCF7L2 comprising the N-terminal 54-residue β-catenin binding domain. We made a conservative serine to cysteine mutation in the wild-type TCF7L2 (1–54) to allow us to label the protein at a unique site. The wild-type TCF7L2 (1–54) construct contains eight serine residues, excluding the one left behind as a result of removing the purification tag with thrombin. The crystal structures were inspected to find a position where the addition of a bulky, hydrophobic dye molecule would not have a significant steric effect on binding but would be able to report on complex formation. A contact map analysis revealed that only one serine residue, S^31^, formed no contacts to β-catenin in any of the crystal structures, indicating that it is in a sterically unhindered position. Consequently, S^31^ was mutated to a cysteine for labelling with fluorescein maleimide at this position. The TCF7L2 (1–54) S31C construct is hereafter referred to as “WT” and is used as a pseudo-wild type with which all other TCF7L2 mutants are compared. In order to determine whether the S31C mutation and its subsequent labelling with fluorescein maleimide had a significant effect on TCF7L2 binding to β-catenin, equilibrium binding experiments were performed using ITC. Previous studies have used ELISA [61] and ITC [29] to determine the binding affinities for β-catenin and similar TCF7L2 fragments. The values they obtained were 15 ± 6 nM and 16 ± 3 nM for TCF7L2 (1-53) and TCF7L2 (1-57), respectively. The results of the ITC experiments showed that there was no significant difference between TCF7L2 (1–54), WT, fluorescein-labelled WT, and the previous literature values (Table S1 and Figure S1).

**Figure 3:**
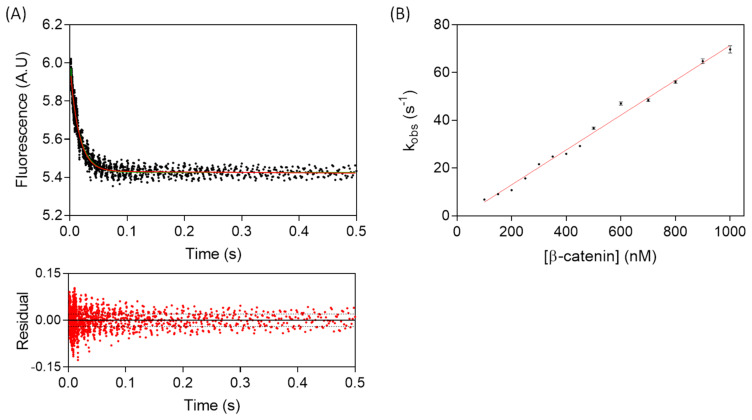
Stopped-flow fluorescence measurement of the association kinetics of TCF7L2 and β-catenin. (**A**) Time-dependent change in fluorescence upon the mixing of 10 nM fluorescent-labelled WT TCF7L2 (1–54) and 900 nM β-catenin. The data were fitted to a single-exponential function, and the residuals are shown below the main plot. (**B**) The observed rate constant was plotted as a function of the concentration of β-catenin, from which the association rate constant, *k_on_*, was calculated. The errors bars in (**B**) are the standard deviations of the fits. Experiments were performed in PBS buffer, 1 mM DTT at 15 °C.

Association kinetics were monitored by the change in fluorescence intensity of the conjugated fluorescein moiety in a stopped-flow fluorimeter. Dissociation kinetics were monitored using either a stopped-flow fluorimeter or a fluorescence spectrometer, depending on the timescale of the reaction. Association kinetics were initiated by rapid mixing in the stopped-flow of solutions containing a fixed concentration of fluorescein-labelled WT, as well as varying concentrations of β-catenin, in order to produce a pseudo-first-order regime. All of the association traces showed a mono-exponential decrease in fluorescence with time, indicating a single association event (Figure 3). Additionally, the observed association rate (*k_obs_*) linearly increased with increasing β-catenin concentration, thus confirming that the reaction is bimolecular. The association rate constant, *k_on_*, calculated from the pseudo-first-order plot, was 7.33 ± 0.14 × 10^7^ M^−1^·s^−1^.

**Figure 4:**
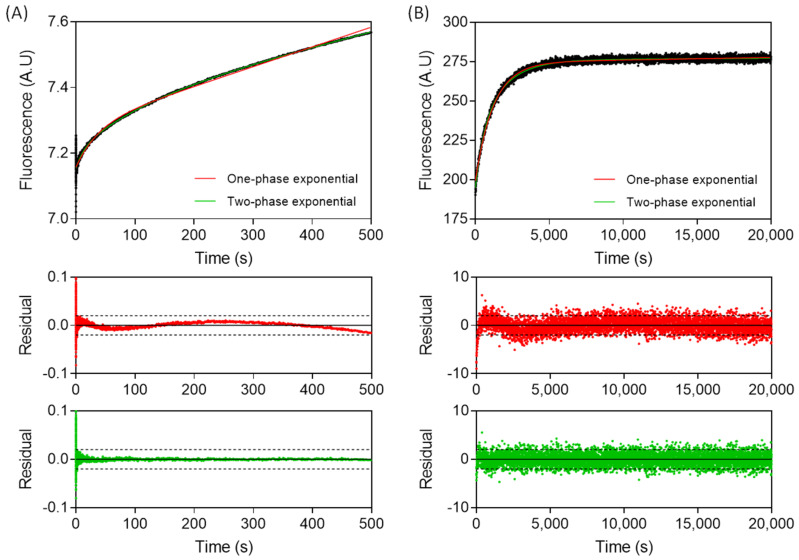
Stopped-flow and time-dependent fluorescence spectroscopy measurements of the dissociation of the WT TCF7L2–β-catenin complex. First, a 200 nM fluorescent-labelled WT TCF7L2–β-catenin complex was mixed in with 50-times molar excess of unlabelled WT TCF7L2 (1–54), and the reaction was monitored by (**A**) stopped-flow fluorescence and (**B**) fluorescence. The traces were fitted to a single exponential phase and to the sum of two exponential phases, and the corresponding residuals are plotted below the main plot. Experiments were performed in a PBS buffer, 1 mM DTT at 15 °C.

Dissociation kinetics were monitored by first forming a 1:1 complex of β-catenin and fluorescent-labelled WT TCF7L2. The complex was dissociated by mixing with excess unlabelled TCF7L2 (1–54). Dissociation traces showed a biphasic increase in fluorescence intensity, demonstrating that, unlike association, dissociation is a two-step process (Figure 4). The traces did not reach a plateau when monitored by stopped flow (Figure 4A), and a fluorimeter was consequently used instead (Figure 4B). The data were fitted to the sum of two exponential phases, comprising a major slow phase with *k_off,major_* = 5.73 ± 0.40 × 10^−4^ s^−1^ (relative amplitude of 77 ± 3%) and a minor fast phase with *k_off,minor_* = 1.52 ± 0.28 × 10^−3^ s^−1^ (relative amplitude of 23 ± 3%). The dissociation kinetics were independent of β-catenin concentration.

### 3.2. Alanine Scanning of TCF7L2 Reveals Three Distinct Interface Regions

To investigate the contribution of individual residues to the kinetics of the TCF7L2–β-catenin interaction, an alanine scan of TCF7L2 was performed. Residues spanning the length of the β-catenin binding interface were mutated to alanine. The association traces of all of the mutants showed a single-exponential decrease in fluorescence intensity, similar to that of WT, and none had a significant effect on the association rate constant (Table 1). Several mutants had a very large effect on the dissociation kinetics (Table 1). For I19A, the dissociation kinetics were sufficiently fast to be measurable on the stopped-flow instrument and the fluorimeter. For I19A, F21A, L41A, and V44A, the dissociation kinetics were too fast for the fluorimeter, and the stopped flow was used instead. For all mutants except I19A and F21A, which are discussed in more detail below, the dissociation traces were biphasic with a major slow phase of 70–90% and a minor fast phase of 10–30%, similar to those of WT.

**Figure 5:**
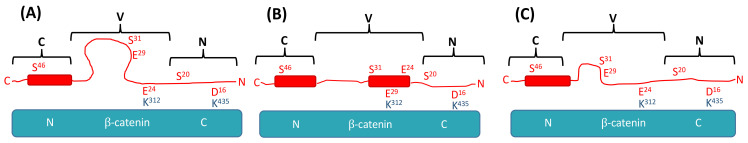
Schematic showing the three interface regions (N, variable (V), and C) of TCF7L2. The schematics show the conformational preferences of TCF7L2 (red) in complex with β-catenin (cyan) in the different crystal structures. (**A**–**C**) are based on the crystal structures of Poy et al. (PDB 1JPW, [22]), Graham et al. (1JDH, [20]), and Sampietro et al. (2GL7, [25])*,* respectively. The N-terminal binding domain (N) and the C-terminal binding domain (C) each adopt very similar conformations in all three structures, but the variable region (V) had a different conformation in each structure.

From these data, we were able to divide TCF7L2 into three distinct regions, which we refer to as the N-terminal binding region (residues 12–22), the variable region (residues 23–39), and the C-terminal binding region (residues 40–50) (Figure 5). Mutations in the N- and C-terminal binding regions had a much larger effect on the TCF7L2–β-catenin kinetics than mutations within the variable region, and a similar trend was previously observed for the equilibrium binding [61,62]. The contributions of the three regions to binding appeared to mirror their conformational properties in crystallo in that the N- and C-terminal regions, which contribute most to the binding affinity, adopted a relatively ‘fixed’ conformation that was similar in all three crystal structures, whereas the variable region was more flexible or ‘pliable,’ adopted different conformations in the three structures, and made little contribution to binding affinity.

### 3.3. Multi-Site Mutations in the Variable Region of TCF7L2 Do Not Perturb the Kinetics

Next, we sought to further explore the role of the variable region. The Sampietro and Poy structures [22,25] both showed Glu24 of TCF7L2 interacting with Lys312 of β-catenin (Figure 5A,C). In the Graham structure [20], it was Glu29 that interacted with Lys312; this difference appeared to be due to residues 21–32 of TCF7L2 forming an α-helix rather than an extended conformation, forcing Glu24 to point away from the surface of β-catenin and positioning Glu29 in the equivalent position where it could interact with Lys312 (Figure 5B). Other studies of β-catenin complexes have identified Lys312 and Lys435 as key interaction residues (often referred to as the “lysine buttons” or “charged buttons”), and so it seems strange that there should have been such variation in the TCF7L2 structure at this critical interface with β-catenin [20,29,63,64]. In the Graham structure, the entire variable region of TCF7L2 (residue 23–39) was resolved, whereas in the other two structures, a significant number of residues were disordered and not visible (residues 27–39 in the Poy structure and residues 29–37 in the Sampietro structure). Additionally, the C-terminal α-helix (residues 41–49), which was present in all three structures, had an extra turn in the Graham structure (residues 37–40). These more extensive helical stretches of the Graham structure had the effect of pulling the variable region across the β-catenin surface. We noticed that there were a further three Glutamate residues close to Glu24 and Glu29 that could potentially interact with Lys312 of β-catenin (Glu23, Glu26, and Glu28), and we postulated that conformations may be populated other than those that are observed in crystallo. By altering the location and length of the N-terminal α-helix of Graham’s structure, the register would be altered and Glu26 or Glu28 could be presented to the lysine button. To investigate this possibility, two additional alanine variants, the double mutant E24A E29A and the quintuple mutant D23A E24A E26A E28A E29A (referred to subsequently 5X), were made. We were surprised to find, given the anticipated key interaction of Glutamate with the lysine button, that neither the double mutant nor the quintuple mutant had a significant effect on the kinetics.

### 3.4. TCF7L2 Mutants I19A and L21A Display More Complex Dissociation Kinetics

**Figure 6:**
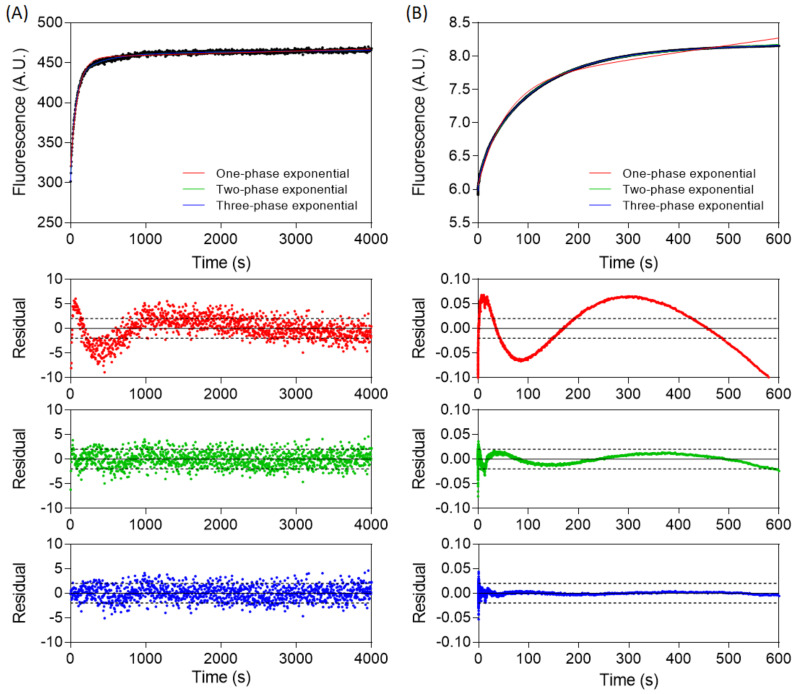
Dissociation kinetics of TCF7L2 I19A. (**A**,**B**) are representative dissociation traces for I19A obtained using the fluorimeter and the stopped-flow instrument, respectively. The data were fitted to one-, two-, and three-phase exponential functions, and the residuals are also shown (red, green, and blue, respectively). Experiments were performed in a PBS buffer, 1 mM DTT at 15 °C.

The dissociation kinetics of the TCF7L2 I19A mutant occurred over a faster timescale than that of WT and could be measured on both the stopped-flow instrument and the fluorimeter (Figure 6). On the stopped flow, the kinetics traces were adequately captured by a biphasic equation with a minor fast phase of 15% of the total amplitude and a major slow phase of 85%, in line with the other mutants. A biphasic equation was also used to fit the kinetic traces obtained with the fluorimeter, with a major fast phase of 82% amplitude and a minor slow phase of 18% amplitude. The slower phase obtained from stopped flow and the faster phase obtained from the fluorimeter had similar rate constants (11.0 × 10^−3^ and 16.5 × 10^−3^ s^−1^, respectively), suggesting that the dissociation of I19A comprises a least three phases in total.

We next attempted to fit the dissociation kinetics of TCF7L2 I19A to a three-phase exponential function, and the obtained rate constants and relative amplitudes are listed in Table 2. Using the fluorimeter, the major phase (75%) had a rate constant of 15.0 ± 0.8 × 10^−3^ s^−1^, and there was a minor (15%) slow phase of 2.7 ± 0.1 × 10^−3^ s^−1^ and a minor (10%) fast phase of 88 ± 19 × 10^−3^ s^−1^. Using the stopped flow, the major phase (79%) was the slowest phase, with a rate constant of 9.1 ± 0.2 × 10^−3^ s^−1^, and there were two faster minor phases with rate constants of 65.2 ± 7.3 × 10^−3^ (15%) and 535 ± 70 × 10^−3^ s^−1^ (16%), as well as a minor middle phase. These results indicated that there were at least three and likely four phases in the dissociation of I19A from β-catenin.

Likewise, for the F21A mutant, the dissociation kinetics measured by stopped-flow were originally fitted to the sum of two exponential phases with a major slow phase (80%) of 25.8 ± 0.2 × 10^−3^ s^−1^ and a minor fast phase (20%) of 423 ± 15 × 10^−3^ s^−1^. However, when these data were fitted to the sum of three exponential phases, there was a major slow phase (66%) of 20.3 ± 0.2 × 10^−3^ s^−1^, a second faster phase (22%) of 91.8 ± 5.9 × 10^−3^ s^−1^, and a third and fastest phase (12%) of 794 ± 68 × 10^−3^ s^−1^. This behaviour was similar to the dissociation kinetics observed for I19A by stopped flow.

In contrast to these two fast-dissociating N-terminal mutants, the kinetics of the two fast-dissociating C-terminal mutants L41A and V44A were biphasic like the WT, and there was no evidence of additional phases.

### 3.5. The Variable Region of TCF7L2 Is Very Sensitive to Ionic Strength

One of the major contributors to binding energy are electrostatic interactions, and their contribution can be investigated by varying the ionic strength of a buffer and measuring the changes in the association and dissociation rate constants. The magnitude of these variations can be assessed from the slopes of the plots of log(*k_on_*) and log(*k_off_*) versus log([NaCl]), referred to as $\Gamma_{NaCl}^{on}$ and $\Gamma_{NaCl}^{off}$, respectively [65,66,67]. A negative value for $\Gamma_{NaCl}^{on}$ and a positive value for $\Gamma_{NaCl}^{off}$ indicate that the complex is destabilised by increasing ionic strength and therefore that electrostatic interactions are involved.

(2) $$\Gamma_{NaCl}^{on}=\frac{\partial \log\left(k_{on}\right)}{\partial \log\left(\left[NaCl\right]\right)}$$

(3) $$\Gamma_{NaCl}^{off}=\frac{\partial \log\left(k_{off}\right)}{\partial \log\left(\left[NaCl\right]\right)}$$

**Figure 7:**
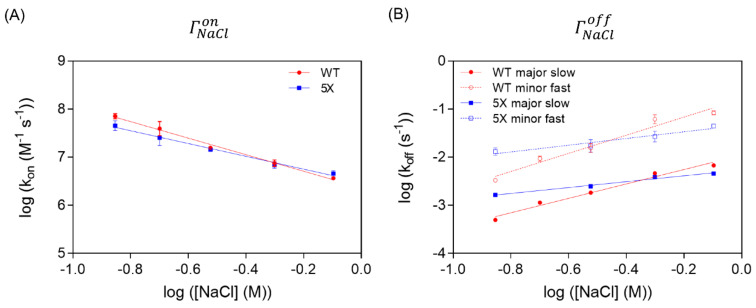
The effect of varying NaCl concentration on the kinetics of the interaction between β-catenin and the WT TCF7L2 and 5X variant. The effects of increasing NaCl concentration on the rate constant for association (**A**) and the rate constants for the two dissociation phases (**B**).

We used this method to test the interaction of β-catenin with both the WT TCF7L2 and the 5X variant, as we thought that the removal of five negatively charged residues might produce a significant change in any electrostatic interactions at the binding interface. We found for both WT and 5X that the association rate decreased with increasing ionic strength and the dissociation rate increased with increasing ionic strength (Figure 7 and Table 3). However, the ionic strength dependence of the WT interaction was greater than that of the 5X mutant, and the 5X mutation had a much larger effect on the ionic strength dependence of dissociation ($\Gamma_{NaCl}^{off\;major}$ and $\Gamma_{NaCl}^{off\;minor}$) than on the salt dependence of association.

## 4. Discussion

Our analysis of the kinetics of the β-catenin–TCF7L2 interaction was dissected by site-directed mutagenesis and uncovered a complex molecular recognition mechanism. We found that there are two key interface regions of TCF7L2—an N-terminal binding region (residues 12–22) and a C-terminal binding region (residues 40–48)—that significantly contribute to the binding to β-catenin, and these are the regions that are the most similar between the different crystal structures. The single-exponential nature of the association kinetics makes it impossible to distinguish which section of this extended interface binds first, but it does suggest that any intermediates are of high energy, thus indicating that that binding is highly co-operative. The trends observed in the alanine scan were consistent with an avidity mechanism of interaction [49] between β-catenin and TCF7L2; the N- and C-terminal binding regions are connected by a flexible linker, each region binding its own cognate site on β-catenin, and once one site is bound, the other region is held close in space to its corresponding binding site.

The central conformationally ‘variable’ region of TCF7L2 (residues 22–40), in which mutation had no effect on either the association or dissociation kinetics, could be regarded as a structurally malleable or “fuzzy” interaction generated by multiple rapidly exchanging high-entropy contacts, eventually leading to a low entropy state [68,69]. This type of fuzzy interaction, which lacks site-specific interactions, was also recently seen for another binding partner of β-catenin, namely the 15-amino acid repeat region of APC [70]. This fuzziness of the binding of different partners involved in balancing the competing pathways of transcription and degradation may allow for faster responses between the different pathways.

**Figure 8:**
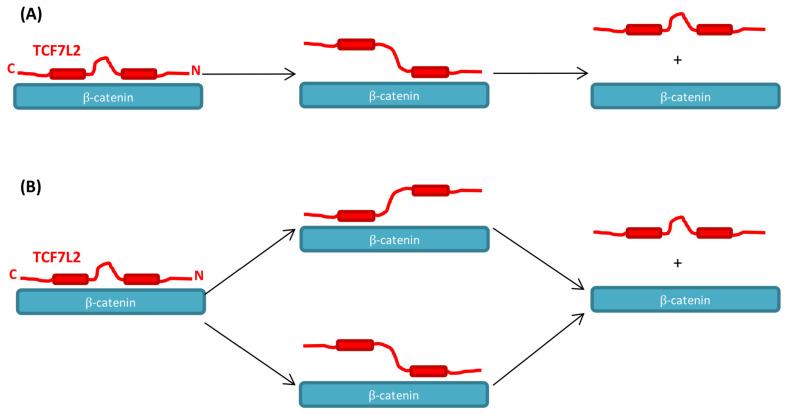
Schematic showing the proposed mechanism of dissociation of TCF7L2–β-catenin complex. TCF7L2 is in red, and β-catenin in cyan. (**A**) shows the sequential dissociation pathway in which the C-terminal region of TCF7L2 always dissociates before the N-terminal region. (**B**) For destabilising N-terminal mutants, there are two parallel dissociation pathways in which either the N-terminal region or the C-terminal region dissociates first.

For the WT and alanine mutants in the C-terminal region of TCF7L2, the dissociation appeared to be biphasic. This behaviour could be explained as arising from sequential dissociation (Figure 8A), whereby the C-terminal region dissociates before the N-terminal region. In contrast, two alanine mutations in the N-terminal region of TC7L2, involving large truncations of hydrophobic sidechains (I19A and F21A), showed a four-phase dissociation reaction. It is likely that the interface-destabilising mutations in the N-terminal region cause a partial shift in flux through an alternative dissociation pathway in which the N-terminal region dissociates before the C-terminal region, resulting in two additional kinetic phases being observed (Figure 8B). This model is also consistent with the observation that the alanine mutants in the C-terminal region generally have a greater effect on the dissociation rate constant than the N-terminal alanine mutants. There are other potential explanations for the biphasic dissociation kinetics, e.g., heterogenous populations of the TCF7L2–β-catenin complex. However, such explanations do not readily explain the consistent site-specific effects of mutations in TCF7L2 (N-terminal versus C-terminal versus middle region) on kinetics.

The general trend for IDP interactions is that increasing ionic strength decreases *k_on_* and has a minimal effect on *k_off_* [51,65,71,72,73]. In contrast, for TCF7L2–β-catenin, there is a roughly equal effect of ionic strength on both dissociation and association—considering either of the two dissociation phases, $\Gamma_{NaCl}^{off}$ is similar in magnitude to $\Gamma_{NaCl}^{on}$. From the sum of $\Gamma_{NaCl}^{on}$ and $\Gamma_{NaCl}^{off}$, we could estimate a value of $\Gamma_{NaCl}^{Kd,\;kin}$≈ −3, which means that the TCF7L2–β-catenin complex is destabilised by three orders of magnitude for every order of magnitude increase in ionic strength. This sensitivity is relatively high compared to other IDP–protein interactions (e.g., $\Gamma_{NaCl}^{Kd,\;kin}$ ≈ −1 for the ACTR–NCBD interaction [72]; −1.4 > $\Gamma_{NaCl}^{Kd,\;kin}$> −2.4 for various HPV E7 constructs binding to Rb [65]). There are parallels between our findings and those for the ACTR–NCBD interaction [72]. When ionic strength was increased, a reversal in the overall charge of NCBD occurred from positive to negative. The explanation given for this effect was that the highly positively charged NCBD attracts a shell of negatively charge chloride ions. At a higher ionic strength, sufficient chloride ions are attracted to this shell to completely screen and eventually overwhelm the positive charge. We believe that a similar behaviour occurs with TCF7L2–β-catenin, with a shell of positively charged sodium ions forming around the negatively charged variable region of TC7L2. The decrease in $\Gamma_{NaCl}^{on}$ upon mutation to the alanine of all five charged residues can be readily explained, as increasing the ionic strength has less of an effect on the interaction with β-catenin. The concept of an ionic shell/screen rationalises the unusually high $\Gamma_{NaCl}^{off}$, as well as the magnitude of the effect of the 5X Glu-to-Ala mutation on $\Gamma_{NaCl}^{off}$. As the variable region fluctuates between different conformations, it periodically dissociates from the surface of β-catenin. When TCF7L2 is separated from β-catenin, the negatively charged residues can recruit a shell of positively charged sodium ions that inhibit re-association and promote complete dissociation. This effect increases in magnitude with increasing ionic strength and is ablated when the five charged residues are mutated to alanine in the 5X construct. Intriguingly the results of the recent single-molecule study of the E-cadherin–β-catenin interaction led to a similar suggestion, whereby there is the continuous detachment and reattachment of local E-cadherin segments [57]. We share the authors’ conclusion of a Velcro-like design of many weak contacts on top of a few persistent interactions.

The alternative pathways of binding and unbinding uncovered here for the ARM repeat protein β-catenin nicely mirror the alternative folding and unfolding pathways previously observed and predicted by our group and others for tandem-repeat proteins [74,75,76,77,78,79]. These phenomena reflect the energy landscapes of the repeating architecture, in which the internal translational symmetry affords multiple paths of similar energies, and, consequently, small perturbations such as conservative mutations are sufficient to shift the flux through each. Such behaviour contrasts with many globular proteins, which often have rather complex topologies. In these proteins, there may only be one way to fold the protein, and therefore even drastic perturbations such as circular permutants can be insufficient to enable flux through any other routes [80,81,82]. In the case of binding, the repeat protein–IDP interface is also strikingly different from most protein–protein interactions studied to date, where there is no internal symmetry; it is consequently unlikely that there will be more than one for association/dissociation. In contrast, here, we looked at a relatively long, extended fragment of TCF7L2 binding to a very long interface comprising 11 repeat motifs. Consequently, the potential for the multiple sub-regions to sequentially ‘zip up’ and in different orders dependent on mutation is great. β-catenin is an important therapeutic target due to its deregulation in many diseases. However, both its long and relatively flat surface, as well as the overlapping nature of its interfaces with favourable and unfavourable partner proteins, have made it difficult to inhibit by conventional techniques. An understanding of the molecular recognition mechanisms and the site-specific dissection of the complex pathways of dissociation described here, will help us to design drugs to target the kinetically accessible interfaces of β-catenin (e.g., the surface that interacts with the C-terminal region of TCF7L2, which dissociates first) and thereby most effectively inhibit β-catenin’s transcriptional activity.

## Figures and Tables

**Table 1:** Association and dissociation kinetics for the interaction of alanine mutants of TCF7L2 with β-catenin. All mutations were constructed in the S31C variant (denoted as WT) and were labelled with fluorescein at this position. Association kinetics were measured on a stopped-flow fluorimeter, and dissociation kinetics were measured on a fluorimeter. * The exceptions are I19A, F21A, L41A, and V44A, for which the data listed for association and dissociation kinetics were obtained with the stopped flow fluorimeter.

TCF7L2 Variant	Association	Dissociation		
	*k_on_* (×10^7^ M^−1^·s^−1^)	*k_off,major_* (×10^−4^ s^−1^)	*k_off,minor_* (×10^−3^ s^−1^)	Relative Amplitude of Minor Phase%
WT	7.33 ± 0.14	5.73 ± 0.40	1.52 ± 0.28	23 ± 3
L12A	7.19 ± 0.13	9.77 ± 0.75	2.64 ± 0.20	19 ± 12
N15A	6.25 ± 0.23	4.42 ± 0.15	3.65 ± 0.83	18 ± 2
E17A	6.30 ± 0.42	89.1 ± 2.9	28.3 ± 1.9	28 ± 4
I19A *	7.04 ± 0.52	110 ± 0.5	207 ± 10	15 ± 0.1
F21A *	4.82 ± 0.17	258 ± 2	423 ± 15	20 ± 0.1
D23A	5.31 ± 0.25	3.00 ± 0.16	2.39 ± 0.70	15 ± 1
E24A	6.05 ± 0.12	5.03 ± 0.40	1.53 ± 0.21	26 ± 1
E26A	5.59 ± 0.22	4.74 ± 0.30	1.66 ± 0.16	24 ± 4
E28A	5.84 ± 0.35	4.96 ± 0.35	1.16 ± 0.28	23 ± 5
E29A	5.73 ± 0.43	8.97 ± 0.44	2.45 ± 0.21	18 ± 2
E38A	6.20 ± 0.19	5.39 ± 0.50	2.61 ± 0.28	30 ± 1
L41A *	5.95 ± 0.24	993 ± 17	616 ± 111	11 ± 0.4
V44A *	5.05 ± 0.46	742 ± 8	498 ± 70	11 ± 0.4
K45A	10.31 ± 0.19	21.9 ± 0.4	15.0 ± 1.3	19 ± 1
L48A	5.67 ± 0.60	13.2 ± 0.1	4.24 ± 0.13	17 ± 0.6
E24A E29A	6.13 ± 0.34	4.25 ± 0.29	1.73 ± 0.14	18 ± 4
D23A E24A E26A E28A E29A (5X)	4.26 ± 0.17	2.95 ± 0.23	2.34 ± 0.33	15 ± 2

**Table 2:** Multi-phase dissociation kinetics of TCF7L2 I19A.

Instrument	*k*_1_ (×10^−3^ s^−1^)	A_1_ (%)	*k*_2_ (×10^−3^ s^−1^)	A_2_ (%)	*k*_3_ (×10^−3^ s^−1^)	A_3_ (%)	*k*_4_ (×10^−3^ s^−1^)	A_4_ (%)
Fluorimeter	-	-	87.6 ± 18.8	10 ± 0.5	15.0 ± 0.8	75 ± 4.5	2.66 ± 0.06	15 ± 4
Stopped flow	535 ± 70	6 ± 0.5	65.2 ± 7.3	15 ± 0.8	9.1 ± 0.2	79 ± 0.3	-	-

**Table 3:** Ionic strength dependence of the interaction between β-catenin and the WT TCF7L2 and 5X variant.

TCF7L2	Salt Dependence		
	$\Gamma_{NaCl}^{on}$	$\Gamma_{NaCl}^{off\;major}$	$\Gamma_{NaCl}^{off\;minor}$
WT	−1.72 ± 0.08	1.50 ± 0.13	1.89 ± 0.20
5X	−1.33 ± 0.07	0.61 ± 0.06	0.70 ± 0.12

## Data Availability

Data are contained within the article or Supplementary Materials.

## Supplementary Materials

The following are available online at https://www.mdpi.com/article/10.3390/biom11060827/s1. Figure S1: ITC analysis of TCF7L2 constructs binding to β-catenin. The top panels show the heat signal obtained from a series of injections of different TCF7L2 into the ITC cell containing β-catenin, and the bottom panels show the binding curves calculated using the one-site fitting model using the Origin software package. (A–C) are TCF7L2 (1–54), “WT,” and the fluorescein-labelled WT, respectively, binding to β-catenin. Experiments were performed in PBS, 1 mM DTT, at 30 °C. Table S1: Dissociation constants of TCF7L2 constructs binding to β-catenin.

## References

[1] Van Der Wal T., Van Amerongen R. (2020). Walking the tight wire between cell adhesion and WNT signalling: A balancing act for β-catenin. Open Biol.

[2] Arce L., Yokoyama N.N., Waterman M.L. (2006). Diversity of LEF/TCF action in development and disease. Oncogene.

[3] Gammons M., Bienz M. (2018). Multiprotein complexes governing Wnt signal transduction. Curr. Opin. Cell Biol..

[4] Mosimann C., Hausmann G., Basler K. (2009). β-Catenin hits Chromatin: Regulation of Wnt Target Gene Activation. Nat. Rev. Mol. Cell Biol..

[5] Willert K., Jones K.A. (2006). Wnt Signaling: Is the Party in the Nucleus?. Genes Dev..

[6] Schaefer K.N., Bonello T.T., Zhang S., Williams C.E., Roberts D.M., McKay D.J., Peifer M. (2018). Supramolecular assembly of the beta-catenin destruction complex and the effect of Wnt signaling on its localization, molecular size, and activity in vivo. PLoS Genet..

[7] Kimelman D., Xu W. (2006). β-catenin Destruction Complex: Insights and Questions from a Structural Perspective. Oncogene.

[8] Wu G., Xu G., Schulman B.A., Jeffrey P.D., Harper J.W., Pavletich N.P. (2003). Structure of a β-TrCP1-Skp1-β-catenin complex: Destruction motif binding and lysine specificity of the SCFβ-TrCP1 ubiquitin ligase. Mol. Cell.

[9] Harris T.J.C., Tepass U. (2010). Adherens Junctions: From Molecules to Morphogenesis. Nat. Rev. Mol. Cell Biol..

[10] Huber A.H., Weis W.I. (2001). The structure of beta-catenin/E-cadherin Complex and the Molecular Basis of Diverse Ligand Recognition by beta-catenin. Cell.

[11] Pokutta S., Weis W.I. (2007). Structure and Mechanism of Cadherins and Catenins in Cell-Cell Contacts. Annu. Rev. Cell Dev. Biol..

[12] Bugter J.M., Fenderico N., Maurice M.M. (2021). Mutations and mechanisms of WNT pathway tumour suppressors in cancer. Nat. Rev. Cancer.

[13] Yoshida R., Kimura N., Harada Y., Ohuchi N. (2001). The Loss of E-cadherin, α- and β-catenin Expression is Associated with Metastasis and Poor Prognosis in Invasive Breast Cancer. Int. J. Oncol..

[14] Conacci-Sorrell M., Zhurinsky J., Ben-Ze’ev A. (2002). The Cadherin-catenin Adhesion System in Signaling and Cancer. J. Clin. Invest..

[15] Herzig M., Savarese F., Novatchkova M., Semb H., Christofori G. (2007). Tumor Progression Induced by the Loss of E-cadherin Independent of β-catenin/Tcf-mediated Wnt Signaling. Oncogene.

[16] Polakis P. (2007). The Many Ways of Wnt in Cancer. Curr. Opin. Genet. Dev..

[17] Huber A.H., Nelson W.J., Weis W.I. (1997). Three-dimensional Structure of the Armadillo Repeat Region of β-catenin. Cell.

[18] Xing Y., Takemaru K.-I., Liu J., Berndt J.D., Zheng J.J., Moon R.T., Xu W. (2008). Crystal Structure of a Full-length β-catenin. Structure.

[19] Eklof Spink K., Fridman S.G., Weis W.I. (2001). Molecular Mechanisms of β-catenin Recognition by Adenomatous Polyposis Coli Revealed by the Structure of an APC-β-catenin Complex. EMBO J..

[20] Graham T.A., Weaver C., Mao F., Kimelman D., Xu W. (2000). Crystal Structure of a β-catenin/Tcf Complex. Cell.

[21] Ha N.C., Tonozuka T., Stamos J.L., Choi H.J., Weis W.I. (2004). Mechanism of phosphorylation-dependent binding of APC to β-catenin and its role in β-catenin degradation. Mol. Cell.

[22] Poy F., Lepourcelet M., Shivdasani R.A., Eck M.J. (2001). Structure of a Human Tcf4-β-catenin Complex. Nat. Struct. Biol..

[23] Xing Y., Clements W.K., Le Trong I., Hinds T.R., Stenkamp R., Kimelman D., Xu W. (2004). Crystal Structure of a β-catenin/APC Complex Reveals a Critical Role for APC Phosphorylation in APC Function. Mol. Cell.

[24] Pettersen E.F., Goddard T.D., Huang C.C., Couch G.S., Greenblatt D.M., Meng E.C., Ferrin T.E. (2004). UCSF Chimera - A Visualization System for Exploratory Research and Analysis. J. Comput. Chem..

[25] Sampietro J., Dahlberg C.L., Cho U.S., Hinds T.R., Kimelman D., Xu W. (2006). Crystal Structure of a β-catenin/BCL9/Tcf4 Complex. Mol. Cell.

[26] Xing Y., Clements W.K., Kimelman D., Xu W. (2003). Crystal Structure of a β-catenin/Axin Complex Suggests a Mechanism for the β-catenin Destruction Complex. Genes Dev..

[27] Graham T.A., Clements W.K., Kimelman D., Xu W. (2002). The Crystal Structure of the β-catenin/ICAT Complex Reveals the Inhibitory Mechanism of ICAT. Mol. Cell.

[28] Choi H.-J., Gross J.C., Pokutta S., Weis W.I. (2009). Interactions of Plakoglobin and β-catenin with Desmosomal Cadherins. J. Biol. Chem..

[29] Sun J., Weis W.I. (2011). Biochemical and structural characterization of β-catenin interactions with nonphosphorylated and CK2-phosphorylated Lef-1. J. Mol. Biol..

[30] Cadigan K.M., Waterman M.L. (2012). TCF/LEFs and Wnt Signaling in the Nucleus. Cold Spring Harb. Perspect. Biol..

[31] Schuijers J., Mokry M., Hatzis P., Cuppen E., Clevers H. (2014). Wnt-induced transcriptional activation is exclusively mediated by TCF/LEF. EMBO J..

[32] Tang W., Dodge M., Gundapaneni D., Michnoff C., Roth M., Lum L. (2008). A Genome-wide RNAi Screen for Wnt/β-catenin Pathway Components Identifies Unexpected Roles for TCF Transcription Factors in Cancer. Proc. Natl. Acad. Sci. USA.

[33] Hazra A., Fuchs C.S., Chan A.T., Giovannucci E.L., Hunter D.J. (2008). Association of the TCF7L2 Polymorphism with Colorectal Cancer and Adenoma Risk. Cancer Causes Control.

[34] Slattery M.L., Folsom A.R., Wolff R., Herrick J., Caan B.J., Potter J.D. (2008). Transcription Factor 7-like 2 Polymorphism and Colon Cancer. Cancer Epidemiol. Biomarkers Prev..

[35] Wenzel J., Rose K., Haghighi E.B., Lamprecht C., Rauen G., Freihen V., Kesselring R., Boerries M., Hecht A. (2020). Loss of the nuclear Wnt pathway effector TCF7L2 promotes migration and invasion of human colorectal cancer cells. Oncogene.

[36] Vaquero A.R., Ferreira N.E., Omae S.V., Rodrigues M.V., Teixeira S.K., Krieger J.E., Pereira A.C. (2012). Using Gene-network Landscape to Dissect Genotype Effects of TCF7L2 Genetic Variant on Diabetes and Cardiovascular Risk. Physiol. Genomics.

[37] Zhang C., Bao W., Rong Y., Yang H., Bowers K., Yeung E., Kiely M. (2013). Genetic Variants and the Risk of Gestational Diabetes Mellitus: A Systematic Review. Hum. Reprod. Update.

[38] Borgia A., Borgia M.B., Bugge K., Kissling V.M., Heidarsson P.O., Fernandes C.B., Sottini A., Soranno A., Buholzer K.J., Nettels D. (2018). Extreme disorder in an ultrahigh-affinity protein complex. Nature.

[39] Csizmok V., Follis A.V., Kriwacki R.W., Forman-Kay J.D. (2016). Dynamic Protein Interaction Networks and New Structural Paradigms in Signaling. Chem. Rev..

[40] Holmstrom E.D., Liu Z., Nettels D., Best R.B., Schuler B. (2019). Disordered RNA chaperones can enhance nucleic acid folding via local charge screening. Nat. Commun..

[41] Van Der Lee R., Buljan M., Lang B., Weatheritt R.J., Daughdrill G.W., Dunker A.K., Fuxreiter M., Gough J., Gsponer J., Jones D.T. (2014). Classification of Intrinsically Disordered Regions and Proteins. Chem. Rev..

[42] Schuler B., Borgia A., Borgia M.B., Heidarsson O., Holmstrom E.D., Nettels D., Sottini A. (2020). Binding without folding-the biomolecular function of disordered polyelectrolyte complexes This review comes from a themed issue on Folding and binding. Curr. Opin. Struct. Biol..

[43] Shammas S.L. (2017). Mechanistic roles of protein disorder within transcription. Curr. Opin. Struct. Biol..

[44] Tsafou K., Tiwari P.B., Forman-Kay J.D., Metallo S.J., Toretsky J.A. (2018). Targeting Intrinsically Disordered Transcription Factors: Changing the Paradigm. J. Mol. Biol..

[45] Uversky V.N. (2011). Multitude of Binding Modes Attainable by Intrinsically Disordered Proteins: A Portrait Gallery of Disorder-based Complexes. Chem. Soc. Rev..

[46] Mittag T., Kay L.E., Forman-Kay J.D. (2009). Protein Dynamics and Conformational Disorder in Molecular Recognition. J. Mol. Recognit..

[47] Borg M., Mittag T., Pawson T., Tyers M., Forman-Kay J.D., Chan H.S. (2007). Polyelectrostatic Interactions of Disordered Ligands Suggest a Physical Basis for Ultrasensitivity. Proc. Natl. Acad. Sci. USA.

[48] Sigalov A.B., Kim W.M., Saline M., Stern L.J. (2008). The Intrinsically Disordered Cytoplasmic Domain of the T Cell Receptor ζ Chain Binds to the Nef Protein of Simian Immunodeficiency Virus without a Disorder-to-order Transition?. Biochemistry.

[49] Olsen J.G., Teilum K., Kragelund B.B. (2017). Behaviour of Intrinsically Disordered Proteins in Protein-Protein Complexes with an Emphasis on Fuzziness. Cell. Mol. Life Sci..

[50] Bachmann A., Wildemann D., Praetorius F., Fischer G., Kiefhaber T., Baldwin R. (2011). Mapping backbone and side-chain interactions in the transition state of a coupled protein folding and binding reaction. Proc. Natl. Acad. Sci. USA.

[51] Dogan J., Gianni S., Jemth P. (2014). The Binding Mechanisms of Intrinsically Disordered Proteins. Phys. Chem. Chem. Phys..

[52] Karlsson E., Paissoni C., Erkelens A.M., Tehranizadeh Z.A., Sorgenfrei F.A., Andersson E., Ye W., Camilloni C., Jemth P. (2020). Mapping the transition state for a binding reaction between ancient intrinsically disordered proteins. J. Biol. Chem..

[53] Rogers J.M., Wong C.T., Clarke J. (2014). Coupled Folding and Binding of the Disordered Protein PUMA Does Not Require Particular Residual Structure. J. Am. Chem. Soc..

[54] Wright P.E., Dyson H.J. (2009). Linking folding and binding. Curr. Opin. Struct. Biol..

[55] Graham T.A., Ferkey D.M., Mao F., Kimelman D., Xu W. (2001). Tcf4 can Specifically Recognize β-catenin using Alternative Conformations. Nat. Struct. Biol..

[56] Daniels D.L., Weis W.I. (2002). ICAT inhibits β-catenin binding to tcf/lef-family transcription factors and the general coactivator p300 using independent structural modules. Mol. Cell.

[57] Wiggers F., Wohl S., Dubovetskyi A., Rosenblum G., Zheng W., Hofmann H. (2021). Diffusion of the disordered E-cadherin tail on β -catenin tail. bioRxiv.

[58] Miroux B., Walker J.E. (1996). Over-production of proteins in Escherichia coli: Mutant hosts that allow synthesis of some membrane proteins and globular proteins at high levels. J. Mol. Biol..

[59] Gasteiger E., Hoogland C., Gattiker A., Duvaud S., Wilkins M.R., Appel R.D., Bairoch A., Walker J.M. (2005). The Proteomics Protocols Handbook—Chapter 52: Protein Identification and Analysis Tools on the ExPASy Server.

[60] Vivès E., Lebleu B. (2003). One-pot labeling and purification of peptides and proteins with fluorescein maleimide. Tetrahedron Lett..

[61] Omer C.A., Miller P.J., Diehl R.E., Kral A.M. (1999). Identification of Tcf4 Residues Involved in High-Affinity β-catenin Binding. Biochem. Biophys. Res. Commun..

[62] Knapp S., Zamai M., Volpi D., Nardese V., Avanzi N., Breton J., Plyte S., Flocco M., Marconi M., Isacchi A. (2001). Thermodynamics of the high-affinity interaction of TCF4 with β-catenin. J. Mol. Biol..

[63] Gail R., Frank R., Wittinghofer A. (2005). Systematic Peptide Array-based Delineation of the Differential β-catenin Interaction with Tcf4, E-cadherin, and Adenomatous Polyposis Coli. J. Biol. Chem..

[64] Xu W., Kimelman D. (2007). Mechanistic Insights from Structural Studies of β-catenin and Its Binding Partners. J. Cell Sci..

[65] Chemes L.B., Sánchez I.E., de Prat-Gay G. (2011). Kinetic Recognition of the Retinoblastoma Tumor Suppressor by a Specific Protein Target. J. Mol. Biol..

[66] Grucza R.A., Bradshaw J.M., Mitaxov V., Waksman G. (2000). Role of Electrostatic Interactions in SH2 Domain Recognition: Salt-Dependence of Tyrosyl-Phosphorylated Peptide Binding to the Tandem SH2 Domain of the Syk Kinase and the Single SH2 Domain of the Src Kinase. Biochemistry.

[67] Vindigni A., White C.E., Komives E.A., Di Cera E. (1997). Energetics of Thrombin−Thrombomodulin Interaction. Biochemistry.

[68] Fuxreiter M. (2020). Classifying the binding modes of disordered proteins. Int. J. Mol. Sci..

[69] Toto A., Malagrinò F., Visconti L., Troilo F., Pagano L., Brunori M., Jemth P., Gianni S. (2020). Templated folding of intrinsically disordered proteins. J. Biol. Chem..

[70] Rudeen A.J., Douglas J.T., Xing M., McDonald W.H., Lamb A.L., Neufeld K.L. (2020). The 15-Amino Acid Repeat Region of Adenomatous Polyposis Coli Is Intrinsically Disordered and Retains Conformational Flexibility upon Binding β-Catenin. Biochemistry.

[71] Hemsath L., Dvorsky R., Fiegen D., Carlier M.F., Ahmadian M.R. (2005). An electrostatic steering mechanism of Cdc42 recognition by Wiskott-Aldrich syndrome proteins. Mol. Cell.

[72] Japrung D., Dogan J., Freedman K.J., Nadzeyka A., Bauerdick S., Albrecht T., Kim M.J., Jemth P., Edel J.B. (2013). Single-Molecule Studies of Intrinsically Disordered Proteins Using Solid-State Nanopores. Anal. Chem..

[73] Rogers J.M., Steward A., Clarke J. (2013). Folding and Binding of an Intrinsically Disordered Protein: Fast, but Not ‘Diffusion-limited’. J. Am. Chem. Soc..

[74] Ferreiro D.U., Cho S.S., Komives E.A., Wolynes P.G. (2005). The energy landscape of modular repeat proteins: Topology determines folding mechanism in the ankyrin family. J. Mol. Biol..

[75] Ferreiro D.U., Walczak A.M., Komives E.A., Wolynes P.G. (2008). The Energy Landscapes of Repeat-Containing Proteins: Topology, Cooperativity, and the Folding Funnels of One-Dimensional Architectures. PLoS Comput. Biol..

[76] Hutton R.D., Wilkinson J., Faccin M., Sivertsson E.M., Pelizzola A., Lowe A.R., Bruscolini P., Itzhaki L.S. (2015). Mapping the Topography of a Protein Energy Landscape. J. Am. Chem. Soc..

[77] Lowe A.R., Itzhaki L.S. (2007). Rational redesign of the folding pathway of a modular protein. Proc. Natl. Acad. Sci. USA.

[78] Tsytlonok M., Sormanni P., Rowling P.J.E., Vendruscolo M., Itzhaki L.S. (2013). Subdomain architecture and stability of a giant repeat protein. J. Phys. Chem. B.

[79] Werbeck N.D., Rowling P.J.E., Chellamuthu V.R., Itzhaki L.S. (2008). Shifting transition states in the unfolding of a large ankyrin repeat protein. Proc. Natl. Acad. Sci. USA.

[80] Lindberg M.O., Oliveberg M. (2007). Malleability of protein folding pathways: A simple reason for complex behaviour. Curr. Opin. Struct. Biol..

[81] Oliveberg M., Wolynes P.G. (2005). The experimental survey of protein-folding energy landscapes. Q. Rev. Biophys..

[82] Otzen D.E., Fersht A.R. (1998). Folding of circular and permuted chymotrypsin inhibitor 2: Retention of the folding nucleus. Biochemistry.

