# Supplementary material for: Parallel and Sequential Pathways of Molecular Recognition of a Tandem-Repeat Protein and Its Intrinsically Disordered Binding Partner
Source: Biomolecules. 2021 Jun 1;11(6):827. doi: 10.3390/biom11060827 (PMC8228192; doi:10.3390/biom11060827)
Supplement: Supplementary file 1 [file biomolecules-11-00827-s001.zip › biomolecules-1203556-supplementary.pdf]

biomolecules

MDPI

Supplementary Information

Table 1. Dissociation constants of TCF7L2 constructs binding to β-catenin.

Construct	Method	K_d (nM)
TCF7L2 (1-53) (Omer *et. al.*)	ELISA	15 ± 6
TCF7L2 (1-57) (Sun & Weis)	ITC	16 ± 3
TCF7L2 (1-54)	ITC	13 ± 7
WT (TCF7L2 (1-54) S31C)	ITC	15 ± 4
Fluorescent-labelled WT	ITC	50 ± 19

Data from Sun and Weis (2011) are for TCF7L2 binding to full-length β-catenin. Experiments were performed in PBS buffer, 1 mM DTT at 30°C, whereas the experiments of Sun and Weis were performed in 25 mM Tris-HCl pH 8.8, 100 mM NaCl, 2 mM DTT at 30°C.

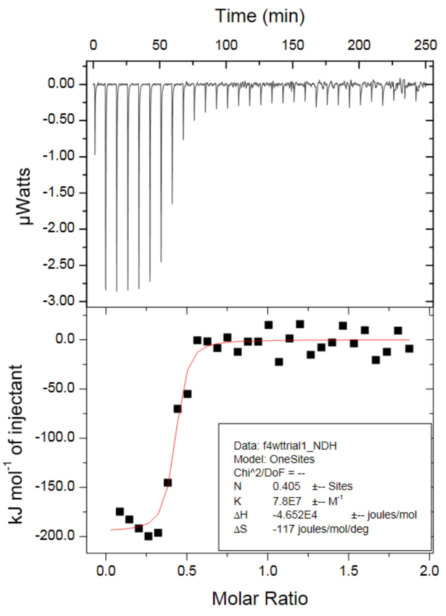

(A)

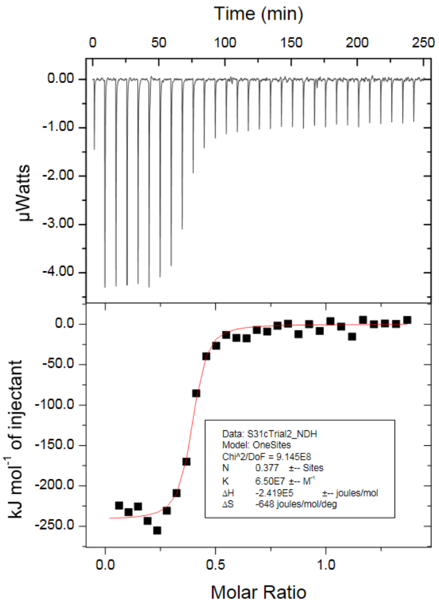

(B)

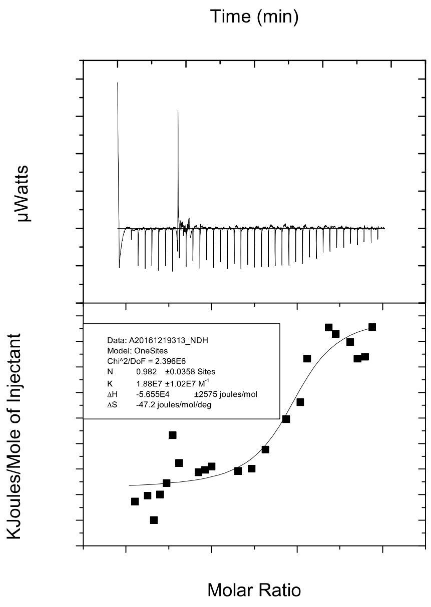

(C)

Figure 1. ITC analysis of TCF7L2 constructs binding to β-catenin. The top panels show the heat signal obtained from a series of injections of different TCF7L2 into the ITC cell containing β-catenin, and the bottom panels show the binding curves calculated using the One-Site fitting model using the Origin software package. (**A**), (**B**) and (**C**) are TCF7L2 (1-54), "WT" and the fluoresce-in-labelled WT, respectively, binding to β-catenin,. Experiments were performed in PBS buffer, 1 mM DTT, at 30°C.
